# Ketamine decreases neuronally released glutamate via retrograde stimulation of presynaptic adenosine A1 receptors

**DOI:** 10.1038/s41380-021-01246-3

**Published:** 2021-08-11

**Authors:** Vesna Lazarevic, Yunting Yang, Ivana Flais, Per Svenningsson

**Affiliations:** Department of Clinical Neuroscience, Karolinska Institutet, Stockholm, Sweden

**Keywords:** Depression, Neuroscience

## Abstract

Ketamine produces a rapid antidepressant response in patients with major depressive disorder (MDD), but the underlying mechanisms appear multifaceted. One hypothesis, proposes that by antagonizing NMDA receptors on GABAergic interneurons, ketamine disinhibits afferens to glutamatergic principal neurons and increases extracellular glutamate levels. However, ketamine seems also to reduce rapid glutamate release at some synapses. Therefore, clinical studies in MDD patients have stressed the need to identify mechanisms whereby ketamine decreases presynaptic activity and glutamate release. In the present study, the effect of ketamine and its antidepressant metabolite, (2R,6R)-HNK, on neuronally derived glutamate release was examined in rodents. We used FAST methodology to measure depolarization-evoked extracellular glutamate levels in vivo in freely moving or anesthetized animals, synaptosomes to detect synaptic recycling ex vivo and primary cortical neurons to perform functional imaging and to examine intracellular signaling in vitro. In all these versatile approaches, ketamine and (2R,6R)-HNK reduced glutamate release in a manner which could be blocked by AMPA receptor antagonism. Antagonism of adenosine A1 receptors, which are almost exclusively expressed at nerve terminals, also counteracted ketamine’s effect on glutamate release and presynaptic activity. Signal transduction studies in primary neuronal cultures demonstrated that ketamine reduced P-T286-CamKII and P-S9-Synapsin, which correlated with decreased synaptic vesicle recycling. Moreover, systemic administration of A1R antagonist counteracted the antidepressant-like actions of ketamine and (2R,6R)-HNK in the forced swim test. To conclude, by studying neuronally released glutamate, we identified a novel retrograde adenosinergic feedback mechanism that mediate inhibitory actions of ketamine on glutamate release that may contribute to its rapid antidepressant action.

## Introduction

Major depressive disorder (MDD) is a leading cause of disability worldwide. The most prescribed antidepressant medications target monoamine neurotransmitter function, but their therapeutic response requires treatment for 2–3 months and they are ineffective in >30% of patients [1]. Contrariwise, a single systemic dose of ketamine, a N-methyl-D-aspartate receptor (NMDAR) antagonist [2] can rapidly reduce suicidal ideation and produce an antidepressant effect even in MDD patients who are resistant to monoamine-based antidepressants [3–5]. *S-*ketamine is approved for clinical use in the form of nasal spray [6]. However, its usage is limited by adverse events which include psychotomimetic and dissociative symptoms along with abuse liability. Understanding the mechanisms of ketamine’s rapid-onset antidepressant action may aid the development of novel antidepressant medications that have fewer side effects.

Experimental data have proved rapid and sustained antidepressant-like activity of (R, S)-ketamine, its two enantiomers, (R)-ketamine and (S)-ketamine as well as its active metabolite (2 R, 6 R)-HNK in rodent models [7–9]. Extensive research has led to development of several models of ketamine’s and (2 R, 6 R)-HNK’s antidepressant mechanisms, involving their postsynaptic action to potentiate BDNF [10], AMPA [11, 12] and dopamine D1 [13, 14] receptor functions. This results in modulation of neuroplasticity pathways, including activation of mTORC1 kinase/eukaryotic initiation factor 4E-binding protein signaling [12, 15] and inhibition of eEF2 kinase [10] along with increased dendritic spine formation [12, 13, 16]. However, few studies have addressed direct effects of ketamine on presynaptic neurotransmission. A critical question in the field is to clarify how ketamine regulates extracellular glutamate levels and whether such regulation is important for its rapid- antidepressant activity [17]. The relevance of this question is strengthened by compelling evidences demonstrating that aberrant glutamatergic signaling associates with pathophysiology of variety of mood disorders, including MDD [18–20]. Most studies report that ketamine exerts its antidepressant activity by blocking NMDARs on GABAergic interneurons and thereby disinhibits afferens to glutamatergic principal neurons resulting in increased extracellular glutamate levels [21–24]. Data for this, so called disinhibition hypothesis, have been generated mainly by in vivo microdialysis [14, 21] and/or NMR spectroscopy [14, 25]. However, a recent study utilizing in vivo microdialysis could not detect a correlation between increased glutamate levels and antidepressant effect by ketamine [26]. Techniques with higher temporal resolution, such as electrophysiological or biosensor recordings, have reported that ketamine increases [27], has no effect [28] or decreases [29] glutamate release. However, the aforementioned techniques are not feasible to perform in MDD patients. A clinical study employing carbon-13 magnetic resonance spectroscopy (^13^C MRS) found increased glutamine upon ketamine administration [30]. In contrast, a more recent MRS study has found that therapeutic actions of ketamine in MDD patients correlates with suppression of glutamate plus glutamine (Glx) in prefrontal cortex [31]. Likewise, ketamine normalizes subgenual cingulate cortex hyper-activity in patients with MDD [32] and reduces brain metabolism [33]. These clinical studies stress the importance of identifying molecular mechanisms whereby ketamine can reduce glutamate levels.

The aim of the present study was to improve mechanistic understanding on rapid effects of ketamine on presynaptic mechanisms underlying neuronal-dependent glutamate release in prefrontal cortex (PFC) and subiculum, two brain regions afflicted in MDD [34, 35]. Several methodological approaches were employed: (1) We studied the local and systemic effects of ketamine and (2 R, 6 R)-HNK in vivo on presynaptic glutamate release in freely moving animals and in anesthetized mice using Fast Analytic Sensing Technology (FAST). FAST is an enzyme-based microelectrode assay coupled with amperometric recordings, that allows to access glutamate level in various brain regions of living animals with very high spatial (<0.2 µM) and temporal resolution (500 ms) [36, 37]. (2) We studied ex vivo synaptic vesicle recycling in synaptosomes from mice systemically treated with of ketamine or (2 R, 6 R)-HNK. (3) Using primary neuronal cultures, we studied synaptic vesicle recycling and presynaptic signal transductions mechanisms in vitro, primarily employing a functional imaging technique based on synaptotagmin1-lumenal domain antibody (syt1-L ab) reuptake assay followed by immunocytochemistry. (4) Signal transduction studies and measures of the phosphorylation state of synapsin, a key presynaptic protein, were made in primary neuronal cultures.

## Materials and methods

### Animals and ethics

All animal work has been done in agreement with the European Council Directive (86/609/EE) and approved by the local Animal Ethics Committee (Stockholms Norra Djurförsöksetiska Nämnd, approval number N24/12; N270/15, N269/13; 1519/2017). An effort was made to reduce the number of animals and to minimize their suffering. For FAST and behavioral analysis 8–10 weeks old male C57Bl/6 J wildtype mice (Charles River, Erkrath, Germany) were used. A series of FAST experiments were also performed in p11 knockout (KO) mice and their wildtype littermates [38]. Mice were kept in a 12 h light–dark cycle with ad libitum food and water. For preparation of primary cortical neurons, E18 pregnant Wistar rats were purchased from Janvier Labs, France.

### Drugs and treatments

Drugs were either intraperitoneally (i.p.) or locally administered into certain brain areas as described in the text. For i.p. injections ketamine and (2 R, 6 R)-HNK were used in a dose of 15 mg/kg that is in a range with doses used in previous studies [9, 10, 12], whereas the final dose used for the local injection during FAST measurement or treatment of primary neurons was determined in dose-dependence experiments and set to 100 µM for both drugs (Supplementary Fig. 1a, b). D-cycloserine was also examined at 100 µM. The list of all pharmacological compounds used in the study is given in Supplementary Materials. Treatment description is notified within figure legends.

### FAST experiments to measure glutamate levels in vivo

FAST recording was essentially done as previously described using either isoflurane anesthetized [29] or freely moving mice [36]. Detailed protocol is given in Supplementary Materials.

### Preparation of synaptosomes and ex vivo studies of synaptic vesicle recycling

Preparation of synaptosomes and their functional assessment was done according to manufacturer’s protocol by using Synaptic Protein Extraction Reagent (Syn-PER) and FM 2–10 membrane probe, respectively (Thermo Fisher Scientific, Waltham, MA, USA).

### Preparation of primary neuronal cultures and in vitro studies of synaptic vesicle recycling and signal transduction

Preparation of primary cortical neurons, Syt1-L ab uptake assay and immunocytochemistry were done as described in Supplementary Information. For the siRNA approach, SMARTpool Accell Adora1 or Accell Non-targeting Pool siRNA were purchased from Dharmaco (Lafayette, CO, USA). The procedure was done according to manufacturer’s protocol. Briefly, primary cortical neurons at DIV14 were transfected either with Adora1 pool siRNA or with non-targeting pool siRNA for 96 h. The knockdown efficacy was evaluated using Dansyl-NECA (Tocris Bioscience, Bristol, UK), a potent and selective fluorescent adenosine A1 agonist. Dansyl-NECA (100 nM) was applied to transfected cells for 30 min and the fluorescence was detected using a Tecan plate reader.

More details on experimental procedures can be found in the Supplementary Information.

## Results

### Ketamine and (2R, 6R)-HNK exert acute reduction of glutamate release in vivo

**Fig. 1:**
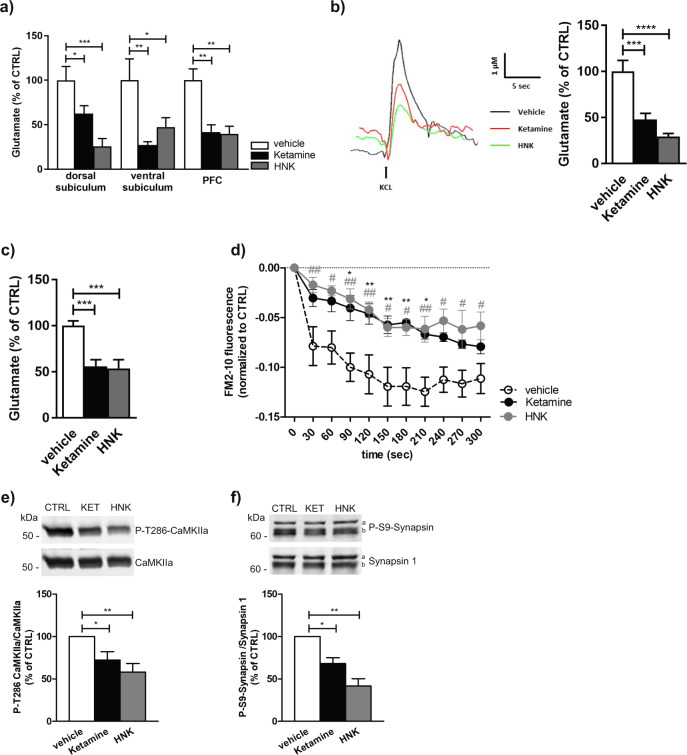
Acute local and systemic administration of ketamine and (2 R, 6 R)-HNK reduces the presynaptic glutamate release. **a** Reduction of KCl-evoked glutamate release upon local application of ketamine or (2 R, 6 R)-HNK (100 µM) into dorsal subiculum, ventral subiculum or prefrontal cortex (PFC) of isoflurane anesthetized mice. Statistical significance was assessed using one-way ANOVA followed by Fisher’s LSD. Dorsal subiculum *N* = 7 animals per each group; *F* (2, 18) = 10.44, *p* = 0.001; ventral subiculum *N* = 6 vehicle; *N* = 5 ketamine; *N* = 6 (2 R, 6 R)-HNK treated mice; *F* = (2, 13) = 5.15, *p* = 0.022; PFC *N* = 6 vehicle; *N* = 6 ketamine; *N* = 5 (2 R, 6 R)-HNK treated animals; *F* (2, 14) = 11.41, *p* = 0.001. *p < 0.05, **p < 0.01, ***p < 0.001. **b** Representative examples of traces and corresponding quantification of KCl-evoked glutamate responses upon local application of vehicle, ketamine or (2 R, 6 R)-HNK (100 µM) into dorsal subiculum of freely moving mice. One-way ANOVA followed by Fisher’s LSD, *N* = 7 animals per each group; *F* (2, 18) = 20.86, *p* < 0.0001. ***p < 0.001 ****p < 0.0001. **c** Reduction of glutamate release 30 min upon systemic administration of ketamine or (2 R, 6 R)-HNK via i.p. injection (15 mg/kg). *N* = 9 vehicle; *N* = 9 Ketamine; *N* = 7 HNK treated mice; *F* (2, 22) = 13.94, *p* = 0.0001; ***p < 0.001. **d** Release of a fluorescent FM2-10 dye upon KCl stimulation of synaptosomes obtained 30 min after i.p. injection of vehicle, ketamine or (2 R, 6 R)-HNK. *N* = 5 animals per each group; Two-way repeated measures ANOVA followed by Bonferroni’s Multiple Comparison Test; interaction *F* (20, 120) = 2.35 *p* = 0.0024; ketamine/HNK *F* = 9.42, *p* = 0.0035; time *F* = 37.55, *p* < 0.0001; *p < 0.05, **p < 0.01 (ketamine vs vehicle); ^#^*p* < 0.05, ^##^*p* < 0.01 (HNK vs vehicle). **e**, **f** Representative western blots and corresponding quantification of pCaMKIIa/CaMKIIa (**e**) and P-S9-Synapsin/Synapsin 1 (**f**). Signal for phospho-protein was normalized to total protein and expressed as % of CTRL for each independent experiment. In (**f**) a denotes Synapsin 1a and b Synapsin 1b. Statistical significance was assessed using one-way ANOVA followed by Bonferroni’s Multiple Comparison Test. In (**e**) data are obtained from *N* = 4 independent synaptosomal preparations. *F* (2, 11) = 13.57, *p* = 0.006; *p < 0.05, **p < 0.01. In (**f**) data originated from *N* = 3 independent synaptosomal preparations. *F* (2, 8) = 40.86, *p* = 0.002; *p < 0.05, **p < 0.01. Within each experimental setup, data were normalized to the mean of the control group and expressed as mean ± SEM.

To examine the effect of (R, S)-ketamine and (2 R, 6 R)-HNK on presynaptic glutamate release we employed FAST technology to directly assess glutamate levels in brain regions associated with MDD. Local application of either ketamine or (2 R, 6 R)-HNK into dorsal or ventral subiculum or prelimbic PFC of isoflurane anesthetized mice elicited significant reduction of KCl-evoked glutamate release (Fig. 1a). The same rapid decrease of presynaptic glutamate was also observed in freely moving animals (Fig. 1b) excluding the possible influence of anesthesia. Likewise, reduced glutamate levels were observed upon systemic ketamine or (2 R, 6 R)-HNK administration. As shown in Fig. 1c antidepressant doses of either ketamine or (2 R, 6 R)-HNK induced reduction of depolarization-evoked glutamate release already 30 min upon administration, suggesting very fast presynaptic effects of both drugs.

To elucidate molecular mechanisms underlying the decrease in extracellular glutamate level, we utilized an ex vivo approach. Accordingly, hippocampal/subiculum synaptosomes were isolated from vehicle or drug treated animals and subsequently subjected to either functional or structural studies. To determine the effect of ketamine and (2 R, 6 R)-HNK on SV recycling, we performed an assay using the styryl dye FM2-10. Observed decay of FM2-10 fluorescence is a measure of activity-dependent SV recycling [39], therefore, the lower decline of the signal from synaptosomes derived from either ketamine or (2 R, 6 R)-HNK treated mice suggested impaired SV recycling efficiency in these animals (Fig. 1d). It is known that one of the important modulators of synaptic transmission is presynaptic CaMKII [40] whose activity could be affected by ketamine treatment [41]. Using synaptosomes derived from ketamine and (2 R, 6 R)-HNK treated animals, we showed that both drugs reduced Thr286-(auto)phosphorylation of CaMKIIa, presumably weakening its activity. In addition, we also showed that both ketamine and (2 R, 6 R)-HNK lowered the level of P-S9-Synapsin (Fig. 1e, f) a residue phosphorylated by CaMKI and/or PKA [42].

### Acute ketamine or (2R, 6R)-HNK treatment attenuates presynaptic potentiation in AMPARs-dependent manner

**Fig. 2:**
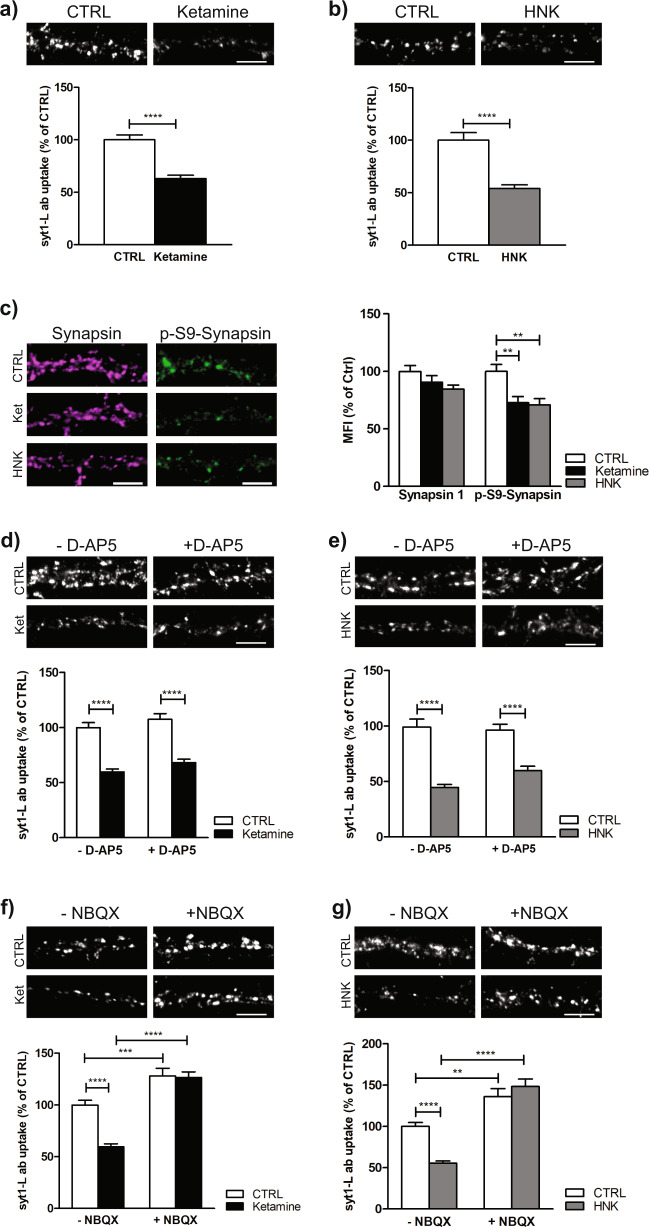
Acute ketamine and (2R,6R)-HNK induced reduction of presynaptic potentiation is AMPAR dependent, but NMDAR independent. **a** Representative images and quantification of KCL-evoked syt1-L ab uptake in CTRL neurons (*N* = 39) and neurons treated for 30 min with 100 µM ketamine (*N* = 46); Student’s *t* test, ****p < 0.0001. **b** Representative images and quantification of KCL-evoked syt1-L ab uptake in CTRL neurons (*N* = 27) and neurons treated for 30 min with 100 µM (2 R, 6 R)-HNK (*N* = 32). Student’s *t* test, ****p < 0.0001. **c** Primary cortical neurons treated for 30 min with CTRL solution (*N* = 30 cells), 100 µM ketamine (*N* = 28 cells) or 100 µM (2 R, 6 R)-HNK (*N* = 32 cells), fixed and stained for total Synapsin 1 (magenta) or P-S9-Synapsin (green). The graph represents a quantification of the signal in corresponding channels. One-way ANOVA followed by Bonferroni’s Multiple Comparison Test. Synapsin 1 F (2, 89) = 2.62; *p* = 0.078; P-S9-Synapsin *F* (2, 89) = 8.47; *p* = 0.0004; **p < 0.01. **d** Representative images and quantification of KCL-evoked syt1-L ab uptake in cells treated for 30 min with 100 µM ketamine in the absence or presence of NMDAR antagonist D-AP5 (30 min; 50 µM) (CTRL *N* = 35; ketamine *N* = 40; D-AP5 *N* = 30; D-AP5/ketamine *N* = 37 cells). Two-way ANOVA with Bonferroni’s Multiple Comparison Test, interaction *F* (1, 138) = 0.02 *p* = 0.89; ketamine *F* = 105.44 *p* < 0.0001; D-AD5 *F* = 4.35 *p* = 0.039; ****p < 0.0001. **e** Representative images and quantification of KCL-evoked syt1-L ab uptake in cells treated for 30 min with 100 µM (2 R, 6 R)-HNK (CTRL *N* = 26: HNK *N* = 32; D-AP5 *N* = 27; D-AP5/HNK *N* = 32 cells) in the absence or presence of NMDAR antagonist D-AP5 (30 min; 50 µM). Two-way ANOVA with Bonferroni’s Multiple Comparison Test, *F* (1, 114) = 3.42 interaction *p* = 0.067; (2 R, 6 R)-HNK *F* = 88.28 *p* < 0.0001; D-AD5 *F* = 1.72 *p* = 0.19; ****p < 0.0001. **f** Representative images and quantification of KCL-evoked syt1-L ab uptake in cells treated with ketamine (30 min; 100 µM) without or with co-treatment with NBQX (30 min; 10 µM) (CTRL *N* = 35; ketamine *N* = 40; NBQX *N* = 34; NBQX/ketamine *N* = 27 cells). Two-way ANOVA with Bonferroni’s Multiple Comparison Test, interaction *F* (1, 132) = 14.25 *p* = 0.0002; ketamine *F* = 16.32 *p* < 0.0001; NBQX *F* = 84.64 *p* < 0.0001; ***p < 0.001, ****p < 0.0001. **g** Representative images and quantification of KCL-evoked syt1-L ab uptake in cells treated with (2 R, 6 R)-HNK in the absence or presence of NBQX (30 min; 10 µM) (CTRL *N* = 25; HNK *N* = 31; NBQX *N* = 34; NBQX/Ketamine *N* = 26 cells). Two-way ANOVA with Bonferroni’s Multiple Comparison Test, interaction *F* (1, 112) = 14.29 *p* = 0.0003; (2 R, 6 R)-HNK *F* = 4.57 *p* < 0.035; NBQX *F* = 73.88 *p* < 0.0001; **p < 0.01, ****p < 0.0001. All data originate from 3 independent cell culture experiments. Bars denote intensity values normalized to the mean intensity value in the control group ± SEM per each experiment. Scale bar 5 µm.

To further evaluate the presynaptic activity following ketamine or (2 R, 6 R)-HNK treatment we employed an in vitro model using rat primary cortical neurons. To assess SV recycling in these neurons, we performed a live staining of both CTRL and drug-treated neurons with fluorophore-coupled antibody recognizing the lumenal domain of the integral SV protein synaptotagmin 1 (syt1-L ab). This antibody binds its target only during the process of exocytosis when SVs fuse with the presynaptic plasma membrane and remain internalized by a compensatory endocytosis. Therefore, the obtained fluorescent signal is directly proportional to presynaptic activity [43]. As shown, acute (5, 15, 30 mins) ketamine or (2 R, 6 R)-HNK treatment attenuates both KCl-evoked (Fig. 2a, b; Supplementary Fig. 2a, b) and network activity driven presynaptic potentiation (Supplementary Fig. 3a, b). In addition, an acute exposure of cells to both drugs also induced long-lasting effects on SV recycling since the reduction in syt1-L ab uptake was preserved 24 h after the drugs were washed out (Supplementary Fig. 4). Taking into account that the majority of synapses in our culture are glutamatergic, we can conclude that both ketamine and (2 R, 6 R)-HNK manifest strong presynaptic properties counteracting glutamate neurotransmission, corroborating in vivo and ex vivo data (Fig. 1). In addition, we also quantified the level of proteins involved in regulation of SV recycling and our data revealed reductions of both P-S9-Synapsin and P-T286-CaMKIIalpha (Fig. 2c; Supplementary Fig. 5a, b). Moreover, at individual synapses, we showed that the level of P-S9-Synapsin correlated well with synaptic activity in CTRL cells and this relationship was also preserved upon drug application (Supplementary Fig. 5c), suggesting that ketamine-induced alterations in presynaptic kinase activites is highly involved in Synapsin 1-mediated regulation of SV recycling.

In addition to ketamine and HNK, D-cycloserine, a partial agonist at the glycine site of NMDA receptors [44] can ameliorate depressive symptomatology [45]. A MRS study showed that D-cycloserine increase Glx in prefrontal cortex [46]. We examined the in vivo regulation of evoked extracellular glutamate levels by D-cycloserine (100 µM) using FAST methodology and found reduced levels (Supplementary Fig. 6a). Likewise, D-cycloserine (10 and 100 µM) reduced synaptic vesicle recycling in primary neuronal cultures (Supplementary Fig. 6b). Since the dose-dependent pharmacology of D-cycloserine at NMDA receptors is complex [44], it is difficult to compare the MRS results [46] and our results. To fully understand the effects of D-cycloserine, time course and dose dependency experiments needs to be done in living animals taking metabolism to bioactive D-serine into account. Nonetheless, these data provide further evidence that a glutamate-based antidepressant agent alter presynaptic glutamate neurotransmission.

Although ketamine acts as a NMDAR antagonist, it was also shown to exhibit NMDAR-independent mode of action that may contribute to its antidepressant activity [11, 47, 48]. In order to test if presynaptic effects of both ketamine and (2 R, 6 R)-HNK require active NMDARs, we performed syt1-L ab uptake assay in cells treated with ketamine or (2 R, 6 R)-HNK in the presence or absence of the NMDAR antagonist, D-AP5. As shown in Fig. 2d, e blockade of NMDARs did not preclude ketamine/(2 R, 6 R)-HNK induced reduction of presynaptic activity, suggesting an alternative mechanism involved in this effects of both drugs. Therefore, we tested the role of AMPA receptors in ketamine/(2 R, 6 R)-HNK induced presynaptic alterations. Our study revealed that the blockade of AMPARs by NBQX increased SV recycling in CTRL cells and completely prevented the inhibitory effect of both ketamine and (2 R, 6 R)-HNK (Fig. 2f, g). Taken together, our data imply that ketamine and (2 R, 6 R)-HNK induced impairment of SV recycling is NMDAR-independent but requires active AMPAR.

### Reduced presynaptic glutamate neurotransmission and antidepressive effects of ketamine and (2R, 6R)-HNK are mediated through AMPA-evoked adenosine signaling

**Fig. 3:**
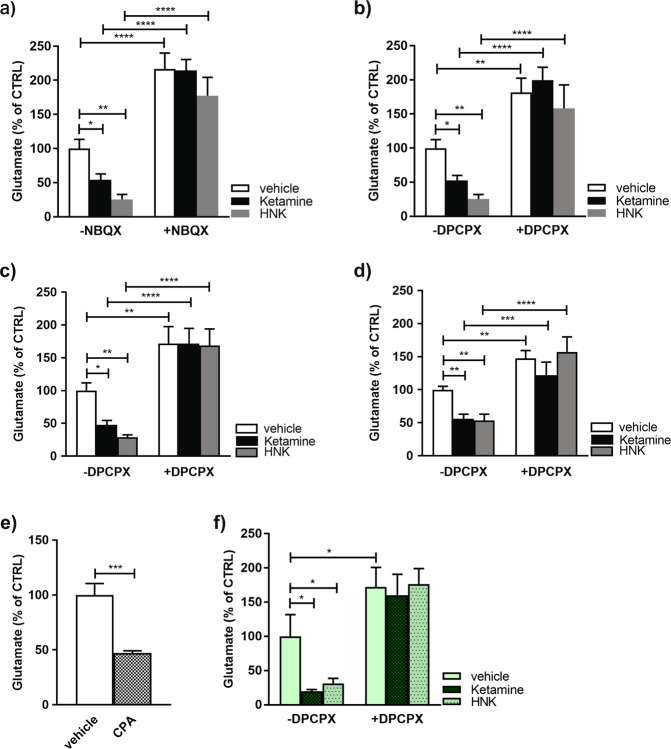
Reduced evoked glutamate release by ketamine and (2 R, 6 R)-HNK is AMPAR-dependent and mediated through A1Rs. **a** Reduction of KCl-evoked glutamate release in subiculum of isoflurane anesthetized mice after local application of 100 µM ketamine or (2 R, 6 R)-HNK was occluded by co-application of NBQX (10 µM). Number of animals per group: *N* = 10 vehicle; *N* = 8 ketamine; *N* = 8 HNK; *N* = 7 NBQX; *N* = 10 NBQX/ketamine; *N* = 5 NBQX/HNK. Statistical significance was assessed by two-way ANOVA followed by Fisher’s LSD; interaction *F* (2, 42) = 1.12, *p* = 0.337; drug *F* = 5.74, *p* = 0.006; NBQX *F* = 116.9, *p* < 0.0001. *p < 0.05, **p < 0.01, ****p < 0.0001. **b** Co-application of DPCPX (2.5 μM) occluded both ketamine and (2 R, 6 R)-HNK induced reduction of KCl-evoked glutamate release in dorsal subiculum of isoflurane anesthetized mice. Number of animals per group: *N* = 9 vehicle; *N* = 9 ketamine; *N* = 9 HNK; *N* = 7 DPCPX; *N* = 8 DPCPX/ketamine; *N* = 7 DPCPX/HNK. Statistical analysis was done using two-way ANOVA followed by Fisher’s LSD; interaction *F* (2, 43) = 1.94, *p* = 0.156; drug *F* = 4.06, *p* = 0.024; DPCPX *F* = 71.36, *p* < 0.0001. *p < 0.05, **p < 0.01, ****p < 0.0001. **c** Reduction of KCl-evoked glutamate release upon local application of 100 µM ketamine or (2 R, 6 R)-HNK into dorsal subiculum of freely moving animals was prevented by co-application of DPCPX (2.5 μM). *N* = 7 vehicle; *N* = 7 ketamine; *N* = 7 HNK; *N* = 6 DPCPX; *N* = 6 DPCPX/ketamine; *N* = 5 DPCPX/HNK treated mice. Two-way ANOVA followed by Fisher’s LSD; interaction *F* (2, 32) = 2.24, *p* = 0.123; drug *F* = 2.52, *p* = 0.096; DPCPX *F* = 65.96, *p* < 0.0001. *p < 0.05, **p < 0.01, ****p < 0.0001. **d** Systemic administration of both ketamine and (2 R, 6 R)-HNK (30 min; i.p. 15 mg/kg) reduced KCl-evoked glutamate release in dorsal subiculum that was prevented by 30 min pre-treatment with DPCPX (2 mg/kg). Number of animals per group: *N* = 9 vehicle; *N* = 9 ketamine; *N* = 7 HNK; *N* = 6 DPCPX; *N* = 6 DPCPX/ketamine; *N* = 5 DPCPX/HNK. Statistical significance was assessed by two-way ANOVA followed by Fisher’s LSD; interaction *F* (2, 36) = 2.63, *p* = 0.085; drug *F* = 4.41, *p* = 0.019; DPCPX *F* = 53.37, *p* < 0.0001. **p < 0.01, ****p < 0.0001. **e** Acute local injection of A1R agonist, CPA (2.5 μM), into dorsal subiculum of isoflurane anesthetized mice significantly reduced KCl-evoked glutamate release. *N* = 10 vehicle; *N* = 8 CPA treated mice. Statistical significance was assessed using Student’s *t* test. ***p < 0.001. **f** Local application of 100 µM ketamine or (2 R, 6 R)-HNK into subiculum of p11 knockout or wildtype mice significantly reduced KCl-evoked glutamate release (*N* = 5 vehicle; *N* = 5 ketamine; *N* = 5 HNK treated animals). Pretreatment of animals with DPCPX (2.5 μM) occluded the effect of both drugs (*N* = 6 DPCPX; *N* = 4 DPCPX/ketamine; *N* = 4 DPCPX/HNK). Two-way ANOVA followed by Fisher’s LSD; interaction *F* (2, 23) = 1.53; *p* = 0.237; drug *F* = 2.042, *p* = 0.153; DPCPX *F* = 36.83, *p* < 0.0001. *p < 0.05.

The AMPAR-dependent reduction of presynaptic glutamate neurotransmission by ketamine and (2 R, 6 R)-HNK was further evaluated in vivo. As seen in Fig. 3a FAST measurement of depolarization-evoked glutamate release clearly disclosed that local application of AMPARs antagonist into subiculum of living mice produced a burst of glutamate in control animals and completely occluded reduction of glutamate release upon ketamine or (2 R, 6 R)-HNK treatments. Activation of both NMDA and AMPA-receptors has been shown to increase extracellular adenosine [49, 50] which, in turn, acts on presynaptic inhibitory A1 or excitatory A2 receptors [51]. Within the brain, A1Rs are widely distributed in cortex, hippocampus, subiculum and cerebellum whereas A2Rs are mostly restricted to striatum and olfactory bulbs [52]. Therefore, we hypothesized that the reduced glutamate levels by ketamine and (2 R, 6 R)-HNK may be mediated through activation of presynaptic A1Rs. To test this hypothesis, we performed both in vivo and in vitro studies using A1R antagonist and agonist, DPCPX and CPA, respectively. Our in vivo data showed that local administration of DPCPX raised extracellular glutamate level and prevented effects of ketamine/(2 R, 6 R)-HNK on glutamate levels in either anesthetized (Fig. 3b) or in freely moving animals (Fig. 3c). A preventive action of DPCPX on sustained ketamine- and (2 R, 6 R)-HNK-mediated reductions of glutamate levels was also detected 24 h post-injections (Supplementary Fig. 7a). Moreover, the same result was also obtained upon i.p. administration of the drugs (Fig. 3d). Conversely, local microinjection of the A1R agonist, CPA, reduced glutamate release similarly to ketamine (Fig. 3e). It is not meaningful to study central actions by systemic administration of CPA as it causes pronounced hypotension and bradycardia [52].

It could be argued that it is more relevant to study effects of ketamine in animals with a depression-like phenotype. We therefore studied the involvement of A1Rs in ketamine/(2 R, 6 R)-HNK induced presynaptic effect using p11KO mice that exhibit a depression-like phenotype [53]. As shown in Fig. 3f and Supplementary Fig. 8, local application of either ketamine or (2 R, 6 R)-HNK into subiculum of p11KO mice markedly attenuated glutamate levels and, similarly to WT animals, this effect was completely abolished by A1R antagonism using DPCPX.

**Fig. 4:**
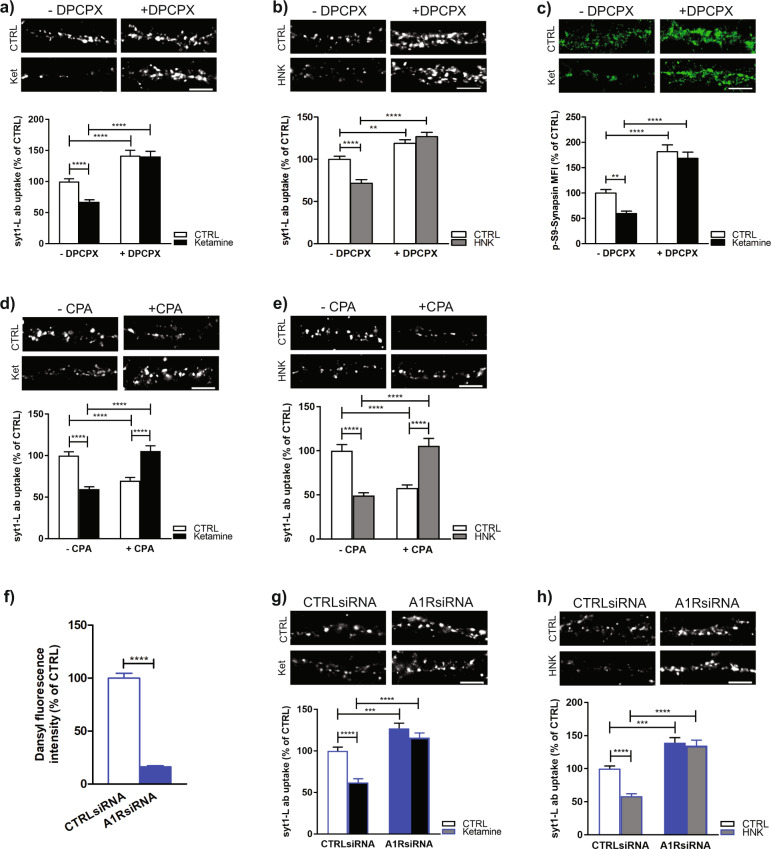
Acute ketamine and (2 R, 6 R)-HNK induced reduction of SVs recycling is mediated via A1R. **a** Representative images and quantification of KCL-evoked syt1-L ab uptake in primary cortical neurons treated with 100 μM ketamine (30 min) without or with pre-treatment with A1R antagonist DPCPX (1 h; 2.5 μM). *N* = 34 CTRL cells; *N* = 36 ketamine; *N* = 36 DPCPX; *N* = 37 DPCPX/ketamine. Two-way ANOVA with Bonferroni’s Multiple Comparison Test, interaction *F* (1, 139) = 5.71 *p* = 0.018; ketamine *F* = 6.64 *p* = 0.011; DPCPX *F* = 75.65 *p* < 0.0001; ****p < 0.0001. **b** Representative images and quantification of KCL-evoked syt1-L ab uptake in primary cortical neurons treated with 100 μM (2 R, 6 R)-HNK (30 min) without or with pre-treatment with A1R antagonist DPCPX (1 h; 2.5 μM). *N* = 31 CTRL cells; *N* = 33 HNK; *N* = 35 DPCPX; *N* = 34 DPCPX/HNK. Two-way ANOVA with Bonferroni’s Multiple Comparison Test, interaction *F* (1, 129) = 19.93 *p* < 0.0001; HNK *F* = 12.03 *p* < 0.0007; DPCPX *F* = 62.99 *p* < 0.0001; **p < 0.01, ****p < 0.0001. **c** Representative images and quantification of P-S9-Synapsin immunofluorescence in CTRL cells or ketamine treated cells (100 μM; 30 min) without or with pre-treatment with DPCPX (1 h; 2.5 μM). Number of cells: CTRL *N* = 35; ketamine *N* = 37; DPCPX *N* = 36; DPCPX/ketamine *N* = 37. Two-way ANOVA with Bonferroni’s Multiple Comparison Test, interaction *F* (1, 141) = 1.88 *p* = 0.173; ketamine *F* = 7.17 *p* = 0.008; DPCPX *F* = 90.63 *p* < 0.0001; **p < 0.01, ****p < 0.0001. **d** KCL-evoked syt1-L ab uptake in primary neurons treated with 100 μM ketamine (30 min) in the absence or presence of an A1R agonist (CPA; 30 min; 2.5 μM). *N* = 35 CTRL cells; *N* = 40 ketamine; *N* = 31 CPA; *N* = 36 CPA/ketamine. Two-way ANOVA with Bonferroni’s Multiple Comparison Test, interaction *F* (1, 138) = 1.88 *p* < 0.0001; ketamine *F* = 0.25 *p* = 0.61; CPA *F* = 3.03 *p* = 0.084; ****p < 0.0001. **e** KCL-evoked syt1-L ab uptake in primary neurons treated with 100 μM (2 R, 6 R)-HNK (30 min) in the absence or presence of an A1R agonist (CPA; 30 min; 2.5 μM). *N* = 27 CTRL cells; *N* = 31HNK; *N* = 29 CPA; *N* = 33 CPA/HNK. Two-way ANOVA with Bonferroni’s Multiple Comparison Test, interaction *F* (1, 116) = 66.83 *p* < 0.0001; HNK *F* = 0.06 *p* = 0.81; CPA *F* = 1.42 *p* = 0.24; ****p < 0.0001. **f** Efficiency of A1R knockdown in primary cortical neurons assessed by Dansyl-NECA fluorescence upon 96 h of transfection using non-targeting (CTRLsiRNA) or Adora1 pool siRNA (A1RsiRNA). Student’s *t* test; ****p < 0.0001. **g**, **h** Absence of A1Rs completely occluded the effect of both ketamine and (2 R, 6 R)-HNK on KCL-evoked syt1-L ab uptake. Ketamine; CTRLsiRNA *N* = 30; CTRLsiRNA/ketamine *N* = 27; A1RsiRNA *N* = 27; A1RsiRNA/ketamine (*N* = 31). Two-way ANOVA with Bonferroni’s Multiple Comparison Test, interaction *F* (1, 111) = 6.47 *p* = 0.012; ketamine *F* = 21.60 *p* < 0.0001; A1RsiRNA *F* = 58.71 *p* < 0.0001; ***p < 0.001 ****p < 0.0001. (2 R, 6 R)-HNK; CTRLsiRNA *N* = 27; CTRLsiRNA/HNK *N* = 30; A1RsiRNA *N* = 29; A1RsiRNA/HNK *N* = 34 on KCL-evoked syt1-L ab uptake. Two -way ANOVA with Bonferroni’s Multiple Comparison Test, interaction *F* (1, 116) = 8.20 *p* = 0.005; HNK *F* = 12.24 *p* = 0.0007; A1RsiRNA *F* = 78.46 *p* < 0.0001; ***p < 0.001 ****p < 0.0001. Data originate from 3 independent cell culture experiments. Bars denote intensity values normalized to the mean intensity value in the control group ± SEM per each experiment.

In accordance with the in vivo data, in vitro experiments revealed that pretreatment of primary neurons with DPCPX increased SV recycling in CTRL cells and completely precluded acute (Fig. 4a, b) as well as sustained (Supplementary Fig. 7b, c) ketamine/(2 R, 6 R)-HNK induced downregulation of syt1-L ab uptake. This was followed by increased P-S9-Synapsin in the presence of DPCPX (Fig. 4c). On the other hand, activation of A1R with CPA reduced SV release probability to a similar extent as ketamine or (2 R, 6 R)-HNK (Fig. 4d, e). However, DPCPX pretreatment could not prevent reduction of presynaptic activity induced by Fluoxetine (Supplementary Fig. 9) suggesting a particularly important role of adenosine signaling in ketamine/(2 R, 6 R)-HNK-mediated actions on presynaptic glutamate neurotransmission.

To further strengthen the hypothesis that A1Rs are involved in ketamine/(2 R, 6 R)-HNK-mediated reduction of presynaptic activity of glutamatergic neurons, we utilized a knockdown approach. Namely, primary cortical neurons were treated with either CTRLsiRNA or A1RsiRNA for 96 h prior to ketamine or (2 R, 6 R)-HNK treatment and followed by syt1-L ab uptake. Knockdown of the target protein was validated using Dansyl-NECA, a potent and selective fluorescent adenosine A1 agonist, that showed almost 80% reduction in cells exposed to A1RsiRNA (Fig. 4f). As shown in Fig. 4g, h the absence of A1Rs completely occluded the effects of both ketamine and (2 R, 6 R)-HNK on presynaptic activity of glutamatergic neurons.

In addition to A1Rs, stimulation of presynaptic cannabinoid receptors (CB1R) also inhibit neurotransmitter release [54]. However, by employing a pharmacological intervention we could not find a significant role of CB1R in ketamine/(2 R, 6 R)-HNK presynaptic action, neither in vivo (Supplementary Fig. 10a) nor in vitro (Supplementary Fig. 10b, c).

Taken together, our data imply that ketamine/(2 R, 6 R)-HNK acting on AMPARs may evoke a postsynaptic adenosine release, which retrogradely feedback onto A1R located on glutamatergic terminals and induce presynaptic inhibition of synaptic recycling and glutamate release.

### Ketamine and (2R, 6R)-HNK exert A1R-dependent rapid antidepressant-like activity in the forced swim test

**Fig. 5:**
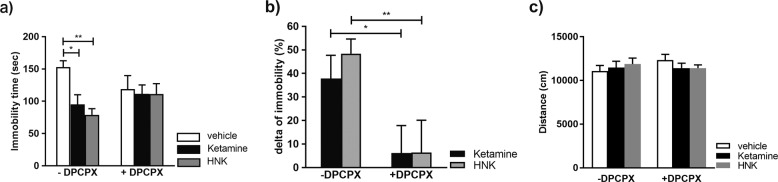
Ketamine and (2R, 6R)-HNK exert A1R-dependent rapid antidepressant-like activity in the forced swim test. **a** Intraperitoneal administration of ketamine or (2 R, 6 R)-HNK (30 min; 15 mg/kg) reduced the immobility time in FST (*N* = 7 vehicle; *N* = 8 ketamine; *N* = 8 HNK mice) that could be prevented by 30 min pre-treatment with 2 mg/kg DPCPX (*N* = 8 DPCPX; *N* = 8 DPCPX/ketamine; *N* = 7 DPCPX/HNK). Statistical significance was assessed using two-way ANOVA followed by Fisher’s LSD; interaction *F* (2, 39) = 2.48, *p* = 0.097; drug *F* = 3.85, *p* = 0.029; DPCPX *F* = 0.142, *p* = 0.708. *p < 0.05, **p < 0.01. **b** Data in (**a**) presented as a delta of immobility and expressed as % of vehicle treated animals. Two-way ANOVA followed by Fisher’s LSD; interaction *F* (1, 27) = 0.239, *p* = 0.629; drug *F* = 0.262, *p* = 0.613; DPCPX *F* = 12.28, *p* = 0.002. *p < 0.05, **p < 0.01. **c** Intraperitoneal administration of ketamine or (2 R, 6 R)-HNK (30 min; 15 mg/kg) does not affect locomotor activity behavior in the open field. Furthermore, there is no difference in total distance with or without 2 mg/kg DPCPX pre-treatment. Number of animals in all groups *N* = 8. Two-way ANOVA followed by Fisher’s LSD; interaction *F* (2, 42) = 1.063, *p* = 0.354; drug *F* = 0.100, *p* = 0.905; DPCPX *F* = 0.251, *p* = 0.619. All data were normalized to the mean of the control group and expressed as mean ± SEM.

Rapid and long-lasting antidepressant-like activity of ketamine and (2 R, 6 R)-HNK in rodents has been demonstrated in the forced swim test (FST) [9–12]. We corroborated these findings by showing that acute (30 min) administration of either ketamine or (2 R, 6 R)-HNK significantly decreased the immobility time compared to saline (Fig. 5a, b). At the same time, neither ketamine nor (2 R, 6 R)-HNK, at given dosages, modified basal locomotor activity (Fig. 5c). Pretreatment of animals with A1R antagonist, DPCPX, occluded effects of both ketamine and (2 R, 6 R)-HNK in the FST, but had no effect on locomotion (Fig. 5a–c). The results provide novel evidence that antidepressant-like effects of ketamine and (2 R, 6 R)-HNK involve functional A1Rs.

## Discussion

The present study was undertaken to elucidate presynaptic mechanisms of rapid antidepressant-like activity of ketamine. Most of previous publications linked acute antidepressant effect of ketamine to drug-induced glutamate surge [21, 30, 55] probably due to selective inhibition of NMDARs located on GABAeric interneurons [14, 24, 56]. However, there are increasing evidences that effects of ketamine on glutamate neurotransmission are regioselective [57] and even synapse specific [27]. Our study, utilizing in vitro and in vivo approaches, showed that both ketamine and (2 R, 6 R)-HNK manifest inhibitory actions on presynaptic glutamate neurotransmission. Namely, local application of either drug into ventral and dorsal subiculum or prelimbic PFC exerted immediate reduction of extracellular glutamate level. Likewise, their systemic administration rapidly reduced brain glutamate level. Appreciating previous findings, it is important to note that most of those studies explored glutamatergic neurotransmission by calculating the glutamate level based on techniques such as microdialysis or magnetic resonance spectroscopy. Neither of them reflect glutamatergic neurotransmission of neurons only, but could also involve important roles of astrocytes known to be critical for glutamate turnover [58]. We employed FAST methodology, with high temporal and spatial resolution that allowed us to estimate presynaptically KCl-evoked released glutamate of entirely neuronal origin [59] in PFC and subiculum. In favor of reduced glutamate release upon ketamine/(2 R, 6 R)-HNK treatment, our in vitro data further provided the evidence that the acute application of both drugs significantly impaired SV recycling, primarily in glutamatergic nerve terminals. These effects could contribute to the efficacy of ketamine to instantly alleviate depressive symptoms and suicidal ideations, taking into account that excessive glutamate levels have been linked to MDD and other mood disorders [20, 60–63]. To study whether the effects of ketamine on glutamate release differed between normal and depression-like mice, we measured glutamate release not only in wildtype mice but also in p11KO mice that exhibit depression-like phenotype in several behavioral paradigms [53]. FAST experiments performed in p11KO mice brought to light that ketamine exerted similar effect on glutamate release in wildtype and p11KO mice.

Although ketamine undoubtedly acts as a NMDAR inhibitor [2] there are evidences indicating that additional mechanisms play important roles for its antidepressant actions [48]. In accordance with previous studies [9, 11, 64, 65] our data revealed AMPARs-mediated effects of both ketamine and (2 R, 6 R)-HNK, suggesting that simple blockade of NMDARs is not sufficient to induce an acute antidepressant response. In fact, dissociative symptoms following ketamine infusion are not associated with its clinical benefits [66], suggesting that NMDAR inhibition may not serve a key role in the antidepressant effects of ketamine. Furthermore, no clinical study in MDD patients have, so far, replicated the full spectrum of rapid and sustained antidepressant actions observed with ketamine using alternative drugs that directly inhibit NMDAR function [67]. On the other hand, our results are supported by preclinical studies showing that AMPARs potentiators may also exert fast antidepressant actions [68, 69]. In addition, activation of AMPARs has been linked to postsynaptically-evoked adenosine release [50, 70] that, in turn, may retrogradely act on presynaptically located adenosine A1Rs and inhibit glutamate release [51, 52]. Using several pharmacological approaches in vitro and in vivo, we firmly demonstrated A1R-mediated presynaptic activity of ketamine and its active metabolite (2 R, 6 R)-HNK. Moreover, by knocking down endogenously expressed A1R in primary neurons, we were able to completely prevent/occlude ketamine/(2 R, 6 R)-HNK-induced reduction of SV recycling, confirming that A1R signaling is, indeed, indispensable for their acute effect on presynaptic potentiation and subsequent glutamate release.

Glutamatergic dysfunction in psychiatric pathophysiology is certainly more complex than the simple designation of glutamate up- or down-regulation, thus, decoding the cellular trigger that initiated ketamine/(2 R, 6 R)-HNK antidepressant response is a key question in the field of antidepressant research. To this end we performed series of experiments to elucidate the molecular mechanisms whereby ketamine/(2 R, 6 R)-HNK and A1-mediated signaling regulate presynaptic glutamate neurotransmission. Several molecules associated with presynaptic release machinery have been involved in depressive disorders and therefore represent valid targets for antidepressant actions [71, 72]. CaMKII is a presynaptically enriched multifunctional enzyme critically involved in regulation of neurotransmitter release through many different mechanisms, including modulation of synapsin-dependent SV translocation from the reserve into the readily releasable pool [40, 73]. Previously it was shown that a single subanesthetic dose of ketamine markedly downregulated Thr286-(auto)phosphorylation of CaMKIIa in hippocampal synaptosomes, that consequently reduced its binding to syntaxin 1 A therefore interfering with SNARE complex assembly [41]. We directly corroborated this finding showing both ketamine- and A1R-mediated reduction of CaMKIIa activity. In addition, we also observed a decreased level of P-S9-Synapsin, that may involve ketamine-induced impairment of other kinases (e.g., PKA and/or CaMKI) whose activity is also important for the regulation of presynaptic release. Increased level of de-phospho synapsin is known to associate with the cytoskeleton and to tie SVs to reserve pool reducing the number of vesicles undergoing exocytosis and therefore lowering presynaptic release potential [73]. Taken together, in accordance with the aforementioned study [41], our data showed ketamine-induced remodeling within presynaptic terminal that may be involved in the molecular mechanisms underlying reduced glutamate release of the drug.

Our study also confirmed a rapid antidepressant effect of ketamine and (2 R, 6 R)-HNK in rodents [9–12]. Namely, at the sub-anesthetic dose of 15 mg/kg both drugs caused a significant decrease in FST immobility already 30 min after their administration, without altering general locomotor activity. Interestingly, treatment with the A1R antagonist, DPCPX, could counteract the effects of ketamine and (2 R, 6 R)-HNK in FST. In accordance with our data, A1R knockout mice display an increased depressive-like behavior [74, 75]. Conversely, transgenic mice with upregulated levels of A1R have pronounced resilience toward depressive-like behavior in various tests [74].

In conclusion, our study sheds a new light on molecular and neurochemical mechanisms underlying the antidepressant effects of ketamine and (2 R, 6 R)-HNK. Both drugs exerted immediate presynaptic response through AMPARs-mediated adenosine release, which retrogradely act on inhibitory A1Rs located on glutamatergic nerve terminals. Activation of these receptors impair propagation of downstream signaling that involves CaMKII and synapsin resulting in impaired redistribution of SVs from the reserve to the readily releasable pool and reduce glutamate release.

## Supplementary information

Supplementary Text

Supplementary Figure 1

Supplementary Figure 2

Supplementary Figure 3

Supplementary Figure 4

Supplementary Figure 5

Supplementary Figure 6

Supplementary Figure 7

Supplementary Figure 8

Supplementary Figure 9

Supplementary Figure 10
