# Supplementary material for: Ketamine decreases neuronally released glutamate via retrograde stimulation of presynaptic adenosine A1 receptors
Source: Mol Psychiatry. 2021 Aug 11;26(12):7425–35. doi: 10.1038/s41380-021-01246-3 (PMC8872981; doi:10.1038/s41380-021-01246-3)
Supplement: Supplementary file 1 — Supplementary Text [file 41380_2021_1246_MOESM1_ESM.docx]

**Ketamine decreases neuronally released glutamate via retrograde stimulation of presynaptic adenosine A1 receptors**

**Vesna Lazarevic, PhD^#^, Yunting Yang PhD^#^, Ivana Flais, MD, Per Svenningsson, MD, PhD***

Department of Clinical Neuroscience, Karolinska Institutet, Stockholm, Sweden

Running title: A1R dependent presynaptic effect of ketamine

# equal contribution

* Corresponding author:

E-mail: Per.Svenningsson@ki.se

Adress: Karolinska Institutet, Department of Clinical Neuroscience, 171 65 Solna, Sweden

Tel: +46-8-1239881

**Supplementary materials and methods**

**Drugs and toxins**

(±)-Ketamine hydrochloride (Sigma-Aldrich; St. Louis, MO, USA); (2R,6R)-Hydroxynorketamine ((2R,6R)-HNK) (Tocris Bioscience, Bristol, UK); D-cycloserine (Selleck chemiclas, Houston, TX, USA); D-AP5 (Tocris Bioscience, Bristol ,UK); NBQX (Tocris Bioscience, Bristol, UK); DPCPX (Tocris Bioscience, Bristol, UK); N6-cyclopentyladenosine (CPA; Tocris Bioscience, Bristol, UK); AM251 (Tocris Bioscience, Bristol, UK); Fluoxetine HCl (LKT Laboratories, St. Paul, MN, USA).

***In vivo* measurement of glutamate release**

Mice were anesthetized with isoflurane (3% for induction, 0.7–1% for maintenance) and mounted in a stereotaxic frame (David Kopf Instruments, Tujunga, CA, USA) fitted with a Cunningham mouse adapter (Stoelting Co. Wood Dale, United States). Small cranial windows were drilled over the recording regions (versus bregma), dorsal subiculum: AP -3.1; ML ±1.5; DV -1.5mm, ventral subiculum: AP -4.3; ML ±3.0; DV -3.0mm, prelimbic prefrontal cortex (PL): AP +1.8; ML ±0.3; DV -2.1mm, and microelectrode arrays (MEAs) were inserted into these regions. As previously described [^1-3^](#_ENREF_1), glutamate dynamics were assessed on a subsecond timescale by MEA recordings with two of four electrode sites coated with L-glutamate oxidase, an enzyme which breaks down L-glutamate into α-ketoglutarate and hydrogen peroxide (H2O2). By using constant voltage amperometry with application of a fixed potential, the H2O2 was oxidized, with electron loss, and the resulting current was recorded using a Fast Analytical Sensing Technology-16 (FAST-16 MKII) electrochemistry instrument (Quanteon LLC, Nicholasville, USA). Depolarization-induced glutamate release was induced by an isotonic solution of 70mM KCl ejected for 1 s at 1 min intervals through a glass micropipette positioned at a distance of 50–100 μm from the MEA recording sites. A MATLAB graphic interface was used to calculate concentrations of glutamate from an average of 3–5 amplitudes per mouse. Maximum amplitude of evoked glutamate release was measured (excluding the amplitudes lower than 0.5 μM). For freely moving recordings, the MEA electrode was implanted into the recording brain region (dorsal subiculum) of the mouse, fixed with dental cement and animal was put back to house cage for 1 week to recover after the surgery. Upon recovery time, mouse was returned to the recording box (PhenoTyper box, Noldus) and glutamate release was measured using the same protocol as for anesthetized mice.

**Isolation of Synaptosomes (synaptoneurosomes)**

Synaptosomes were prepared from the hippocampus of control mice and mice treated with ketamine or HNK. The hippocampal tissue was homogenized using Dounce tissue grinder (10-12 slow strokes) in ice-cold sucrose buffer (320 mM sucrose, 1 mM EDTA, 0.25 mM dithiothreitol, pH 7.4) supplemented with protease and phosphatase inhibitors, Complete mini (Roche, Basel, Switzerland) and PhosStop (Roche, Basel, Switzerland), respectively. The homogenate was centrifuged for 10 min at 1,000 x g in order to remove the cell debris. The supernatant was further centrifuged for 30 minutes at 12,000 x g at 4°C. Obtained pellet (P2 fraction) containing synaptosomes was resuspended in RIPA buffer (25mM Tris, 150mM NaCl, 5mM EDTA, 1% Triton X-100, 1% sodium deoxycholate, 0,1% SDS pH 7,6 supplemented with protease and phosphatase inhibitors) and subjected to Western Blot. For FM2-10 assay, synaptosomes were isolated using Syn-PER Synaptic Protein Extraction Reagent (Thermo Fisher Scientific, [Waltham, MA, USA](https://www.google.com/search?rlz=1C1GGRV_enSE751SE751&sxsrf=ALeKk02JzZjtgPISRRJDIYK9KyLmzr8KVw:1613330901548&q=Waltham,+Massachusetts&stick=H4sIAAAAAAAAAOPgE-LSz9U3MCooMTBJU-IAsTOqjE21tLKTrfTzi9IT8zKrEksy8_NQOFYZqYkphaWJRSWpRcWLWMXCE3NKMhJzdRR8E4uLE5MzSotTS0qKd7AyAgC_w9ZWYAAAAA&sa=X&ved=2ahUKEwiCt6r6jeruAhXmwosKHVcsDz8QmxMoATAaegQIIRAD)) according to manufacturer’s recommendation. Briefly, the tissue was homogenized in Syn-PER buffer supplemented with 1x Complete and PhosStop and centrifuged 10 min at 1,000 x g. The pellet was discarded and supernatant was subsequently centrifuged for 30 minutes at 12,000 x g at 4°C in order to obtain P2 fraction containing synaptosomes. P2 was resuspended in HBSS (with magnesium and calcium, Thermo Fisher Scientific, [Waltham, MA, USA](https://www.google.com/search?rlz=1C1GGRV_enSE751SE751&sxsrf=ALeKk02JzZjtgPISRRJDIYK9KyLmzr8KVw:1613330901548&q=Waltham,+Massachusetts&stick=H4sIAAAAAAAAAOPgE-LSz9U3MCooMTBJU-IAsTOqjE21tLKTrfTzi9IT8zKrEksy8_NQOFYZqYkphaWJRSWpRcWLWMXCE3NKMhJzdRR8E4uLE5MzSotTS0qKd7AyAgC_w9ZWYAAAAA&sa=X&ved=2ahUKEwiCt6r6jeruAhXmwosKHVcsDz8QmxMoATAaegQIIRAD)) and subjected to BCA assay (Thermo Fisher Scientific, [Waltham, MA, USA](https://www.google.com/search?rlz=1C1GGRV_enSE751SE751&sxsrf=ALeKk02JzZjtgPISRRJDIYK9KyLmzr8KVw:1613330901548&q=Waltham,+Massachusetts&stick=H4sIAAAAAAAAAOPgE-LSz9U3MCooMTBJU-IAsTOqjE21tLKTrfTzi9IT8zKrEksy8_NQOFYZqYkphaWJRSWpRcWLWMXCE3NKMhJzdRR8E4uLE5MzSotTS0qKd7AyAgC_w9ZWYAAAAA&sa=X&ved=2ahUKEwiCt6r6jeruAhXmwosKHVcsDz8QmxMoATAaegQIIRAD)) for protein quantification. Equal amounts of synaptosome suspensions were then incubated with 100 µM FM2-10 (Thermo Fisher Scientific, [Waltham, MA, USA](https://www.google.com/search?rlz=1C1GGRV_enSE751SE751&sxsrf=ALeKk02JzZjtgPISRRJDIYK9KyLmzr8KVw:1613330901548&q=Waltham,+Massachusetts&stick=H4sIAAAAAAAAAOPgE-LSz9U3MCooMTBJU-IAsTOqjE21tLKTrfTzi9IT8zKrEksy8_NQOFYZqYkphaWJRSWpRcWLWMXCE3NKMhJzdRR8E4uLE5MzSotTS0qKd7AyAgC_w9ZWYAAAAA&sa=X&ved=2ahUKEwiCt6r6jeruAhXmwosKHVcsDz8QmxMoATAaegQIIRAD)) for 1 min at RT. To stimulate the uptake of the dye, 30 mM KCl was added and the suspension was incubated for another 10 min at RT. Subsequently, the suspension was centrifuged for 5 min at 15,000 x g, supernatant was discarded and the remaining pellet was washed two times with HBSS supplemented with 1 mg/ml BSA in order to remove excess dye. Finally, synaptosomal pellet was resuspended in HBSS and the release of the dye was monitored in the presence of 50 mM KCl by measuring the fluorescence (ex 506/em 620) on a Tecan plate reader (Tecan Life Science, Männedorf, Switzerland).

**Primary cortical neurons**

Primary cortical neurons were prepared from E18 pregnant Wistar rats according to a previously published protocol [^4^](#_ENREF_4). Briefly, the meninges of isolated embryonic cortices were peeled off and tissue was cut into smaller pieces. After several washing steps with ice cold HBSS, tissue was mixed with Neuronal Isolation Enzyme with papain (Thermo Fisher Scientific, [Waltham, MA, USA](https://www.google.com/search?rlz=1C1GGRV_enSE751SE751&sxsrf=ALeKk02JzZjtgPISRRJDIYK9KyLmzr8KVw:1613330901548&q=Waltham,+Massachusetts&stick=H4sIAAAAAAAAAOPgE-LSz9U3MCooMTBJU-IAsTOqjE21tLKTrfTzi9IT8zKrEksy8_NQOFYZqYkphaWJRSWpRcWLWMXCE3NKMhJzdRR8E4uLE5MzSotTS0qKd7AyAgC_w9ZWYAAAAA&sa=X&ved=2ahUKEwiCt6r6jeruAhXmwosKHVcsDz8QmxMoATAaegQIIRAD)) and incubated for 20 min at 37°C. Again extensive washing was performed followed by addition of 0.1% DNAse and disaggregation of the tissue by using syringe and cannulas (3x 20G and 3x26G). Finally, cells were resuspended in warm DMEM (supplemented with FBS, Glutamine and antibiotics) and plated on poly-D-lysine-coated glass coverslips in 24 well plates at a density of 50000/well for ICC or into 6 well plates at a density of 300000/well for western blots. 24h after the plating, DMEM was exchanged with Neurobasal medium (supplemented with B27, glutamine and antibiotics). Cells were kept in a humidified incubator with 5% CO2 for up to 3 weeks and fed with fresh medium every 3^rd^ day.

**Immunocytochemistry and functional imaging**

Synaptotagmin1-L antibody uptake assay was done on primary cells using mouse monoclonal anti-Synaptotagmin 1 lumenal domain antibody (Syt1-L ab) fluorescence-labeled with Oyster 550 (Synaptic Systems, Göttingen, Germany; Cat. No. 105 311C3). Upon the treatment, cells were washed two times with Tyrodes buffer (TB) (in mM: 119 NaCl; 2.5 KCl; 25 Glucose; 2 MgCl2; 2 CaCl2) and stained with Syt1-L ab (1:250) either for 5 min (in TB supplemented with 50 mM KCl) or 20 min (in TB) in order to determine KCl-evoked and network activity driven presynaptic potentiation, respectively. Thereafter, cells were extensively washed with TB in order to eliminate unbound antibody, fixed with 4% PFA for 3 min and blocked/permeabilized for additional 30 min using 10% FBS/0.1% glycin/0.3% Triton-X100 in PBS. Primary antibodies diluted in 3% FBS were applied overnight at 4°C: guinea pig anti-Synaptophysin 1 (1:1000; Synaptic Systems, Göttingen, Germany; Cat. No. 101 004); mouse anti-Synapsin 1 (1:1000; Synaptic Systems, Göttingen, Germany; Cat. No. 106 011); rabbit anti-Phospho-Synapsin (Ser9) (1:500; Cell Signalling, Danvers, MA, USA; Cat. No. 2311). Secondary antibodies diluted also in 3% FBS were applied during 1h at RT: Anti-rabbit Alexa Fluor 488 (Cat. No. A-11008); Anti-mouse Alexa Fluor 568 (Cat. No. A-11031); Anti-guinea pig Alexa Fluor 647 (Cat. No. A-21450). All used in the dilution of 1:1000 (Thermo Fisher, Waltham, MA, USA). Cells were mounted on microscopic glass slides using Mowiol 4-88 (Sigma-Aldrich; St. Louis, MO, USA).

**Western Blot**

Western blot using hippocampal synaptosomes or primary cortical neurons was done according to standard protocol [^5^](#_ENREF_5). Briefly, protein concentration was determined using BCA assay (Thermo Fisher, Waltham, MA, USA) and equal amounts of proteins were loaded onto polyacrylamide gels. In this study the following primary antibodies were used: mouse anti-CaMKII-α (6G9) (1:1000; Cell Signaling, Danvers, MA, USA; Cat. No. 50049); rabbit anti-phospho CaMKII (Thr286) (1:1000; Cell Signaling, Danvers, MA, USA; Cat. No. 12716); rabbit anti-phospho-Synapsin (Ser9) (1:1000; Cell Signalling, Danvers, MA, USA; Cat. No. 2311); mouse anti-Synapsin 1 (1:1000; Synaptic Systems, Göttingen, Germany; Cat. No. 106 011); mouse anti-β-tubulin III (1:1000; Sigma-Aldrich; St. Louis, MO, USA; Cat. No. T8578). Fluorescently labeled secondary antibodies for WB (all 1:20000): anti-rabbit IRDye 800CW (Li-COR Biosciences, Lincoln, NE, USA Cat. No. 926-32211) and anti-mouse IRDye 680RD (Li-COR Biosciences, Lincoln, NE, USA; Cat. No. 926-68070). Membranes were scanned in the appropriate channels using Odyssey CLx Infra Red Imaging system from LI-COR Biosciences (Lincoln, Nebraska USA). Quantification of the signals was done using the software Image Studio 5.1. Upon background subtraction the values for phospho-proteins were normalized to their respective total signals. Data were obtained from ≥4 independent cell culture experiments and ≥3 synaptosomal preparations.

**Image acquisition and analysis**

Images were acquired by ZEISS LSM 880 Airyscan confocal laser scanning microscopy equipped with ZEN2.1 software, using Plan-Apochromat 63x/1.4 Oil DIC M27 63x oil objective. All images from the same experiment were acquired using the same setup. Quantification of the signal was performed using ImageJ (NIH, <http://rsb.info.nih.gov/ij/>) and OpenView software [^6^](#_ENREF_6). Upon background subtraction in ImageJ, synaptic puncta were defined along 20 µm of proximal dendrite by setting a rectangular regions of interest (ROI) in channel positive for Synaptophysin (chosen as synaptic marker) by using OpenView software. Thereafter, mean immunofluorescence intensities (MFI) were measured in the synaptic ROIs in all channels. Data were always normalized to the mean of the control group per each experiment. Final data were obtained from ≥3 independent experiments. For the presentation images were adjusted by using ImageJ and PhotoShop (Adobe Systems).

**Behavioral analyses of mice**

The behavioral studies were assessed by the forced swim test (FST) and open-field test. Briefly, for FST, mice were individually placed in cylindrical tanks (diameter x height: 20×50 cm) filled with water to a depth of 20 cm (water temperature: 24±1 °C). Over a test duration of seven minutes, behavior was recorded and analyzed via an automated video tracking system (Ethovision XT 11.5, Noldus). The open-field test was performed for 30 min in a 46×46 cm^2^ arena with grey floor and walls. The arena was illuminated by reflected light, providing an intensity of 30 Lux on the floor of the arena. The arena was cleaned with 70% ethanol after each test session to eliminate olfactory cues. Video tracking was performed using a video camera mounted in the ceiling and analyzed by EthoVision XT11.5 (Noldus) software.

**Experimental design and Statistics:**

For both *in vivo* and *in vitro* experiments, the sample sizes were based on previous reports to ensure adequate power. For the animal studies, the experimenter was not blinded to group allocation during experiment but the analyses were automated. For the biochemical assays the experimenter was blinded during the experiments and when assessing the outcome. For cell culture experiments the assessment of outcome was blinded. Animals were pseudo-randomly allocated to experimental groups, so each cage contained balanced group distribution. Cell culture data originated from ≥3 independent preparation.

All statistical analysis were done using GraphPad Prism (GraphPad, San Diego, CA, USA). Outlier detection within each data set was assesed using the Grubb’s test calculator tool from GraphPad and identified outliers were removed. Normality distribution of the data was determined using D'Agostino-Pearson normality test in GraphPad Prism. The chosen statistical tests, number of used animals and number of cells analyzed per each group are indicated in figure legends. Within each experimental setup, data were normalized to the mean of the control group and expressed as mean ± SEM. Statistical significance was assessed as *p<0.05; **p<0.01; ***p<0.001; ****p<0.0001.

**Supplementary references**

1. Hascup KN, Hascup ER, Pomerleau F, Huettl P, Gerhardt GA. Second-by-second measures of L-glutamate in the prefrontal cortex and striatum of freely moving mice. *The Journal of pharmacology and experimental therapeutics* 2008; **324**(2)**:** 725-731.

2. Alvarsson A, Zhang X, Stan TL, Schintu N, Kadkhodaei B, Millan MJ *et al.* Modulation by Trace Amine-Associated Receptor 1 of Experimental Parkinsonism, L-DOPA Responsivity, and Glutamatergic Neurotransmission. *The Journal of neuroscience : the official journal of the Society for Neuroscience* 2015; **35**(41)**:** 14057-14069.

3. Stan TL, Alvarsson A, Branzell N, Sousa VC, Svenningsson P. NMDA receptor antagonists ketamine and Ro25-6981 inhibit evoked release of glutamate in vivo in the subiculum. *Translational psychiatry* 2014; **4:** e395.

4. Lazarevic V, Schone C, Heine M, Gundelfinger ED, Fejtova A. Extensive remodeling of the presynaptic cytomatrix upon homeostatic adaptation to network activity silencing. *The Journal of neuroscience : the official journal of the Society for Neuroscience* 2011; **31**(28)**:** 10189-10200.

5. Lazarevic V, Yang Y, Ivanova D, Fejtova A, Svenningsson P. Riluzole attenuates the efficacy of glutamatergic transmission by interfering with the size of the readily releasable neurotransmitter pool. *Neuropharmacology* 2018; **143:** 38-48.

6. Tsuriel S, Geva R, Zamorano P, Dresbach T, Boeckers T, Gundelfinger ED *et al.* Local sharing as a predominant determinant of synaptic matrix molecular dynamics. *PLoS biology* 2006; **4**(9)**:** e271.

**Supplementary Figure legends**

**Supplementary Figure 1. Effect of Ketamine and (2R,6R)-HNK on KCl-evoked glutamate release *in vivo* and syt1-L ab uptake *in vitro* is concentration-dependent. a)** Quantification of KCL-evoked glutamate release in subiculum 30 min after local application of vehicle (N=14), ketamine (in µM: 1 (N=6), 10 (N=8), 30 (N=7), 50 (N=8), 100 (N=7) or (2R,6R)-HNK (in µM: 1 (N=5), 10 (N=5), 30 (N=8), 50 (N=5), 100(N=7)). Statistical significance was assessed using one-way ANOVA followed by Fisher's LSD; F (10, 69) = 3.105, p=0.003. *p<0.05, **p<0.01, ****p<0.0001**. b)** Quantification of KCL-evoked syt1-L ab uptake in primary cortical neurons treated for 30 min with control solution or rising dose of ketamine or (2R,6R)-HNK. CTRL (N=69); ketamine (in µM: 1 (17), 10 (13), 30 (17), 50 (19), 100 (52); CTRL (N=56); (2R,6R)-HNK (in µM: 1 (18), 10 (15), 30 (17), 50 (17), 100 (38). Statistical significance was assessed using one-way ANOVA followed by Bonferroni’s Multiple Comparison Test; F (11, 336) = 17.6, p<0.0001. ***p<0.001. Within each experimental setup, bars denote intensity values normalized to the mean intensity value in the control group ± SEM.

**Supplementary Figure 2. Time course of acute effects of Ketamine and (2R,6R)-HNK on KCl-evoked syt1-L ab uptake *in vitro*. a)** Quantification of KCL-evoked syt1-L ab uptake in primary cortical neurons treated with control solution (N=30) or 100 µM ketamine for 5 min (N=30), 15 min (N=27) and 30 min (N=27). Statistical significance was assessed using one-way ANOVA followed by Bonferroni’s Multiple Comparison Test; F (3, 110) = 16.71, p<0.0001. ****p<0.0001. **b)** Quantification of KCL-evoked syt1-L ab uptake in primary cortical neurons treated with control solution (N=26) or 100 µM (2R,6R)-HNK for 5 (N=27), 15 (N=25) and 30 min (N=26). Statistical significance was assessed using Kruskal-Wallis test followed by Dunn’s Multiple Comparison Test; p<0.0001. ***p<0.001, ****p<0.0001. Within each experimental setup, data were normalized to the mean of the control group and expressed as mean ± SEM.

**Supplementary Figure 3. Reduction of network activity driven (tonic) presynaptic activity upon ketamine and (2R,6R)-HNK treatment of primary cortical neurons.** **a)** Representative images and quantification of tonically driven syt1-L ab uptake in CTRL neurons (N=33) and neurons treated 30 min with 100 µM ketamine (N=33); Student’s t-test, ****p<0.0001. Scale bar 5µm. **b)** Representative images and quantification of tonic syt1-L ab uptake in CTRL neurons (N=35) and neurons treated 30 min with 100 µM (2R,6R)-HNK (N=34); Student’s t-test, ****p<0.0001. Scale bar 5µm. Within each experimental setup, data were normalized to the mean of the control group and expressed as mean ± SEM.

**Supplementary Figure 4. Effect of ketamine and (2R,6R)-HNK on KCl-evoked syt1-L ab uptake *in vitro* is long lasting.** Reduction of KCl-evoked syt1-L ab uptake in cells treated for 30 min with 100 µM Ketamine or (2R,6R)-HNK is preserved 24h after the drugs were washed out (24hWO). N=28 CTRL cells; N=29 ketamine; N=25 HNK; N=29 CTRL/WO; N=29 ketamine/WO; N=26 HNK/WO. Statistical significance was assessed using two-way ANOVA followed by Bonferroni’s Multiple Comparison Test; interaction F (2, 160) = 0.19, p=0.826; drug F=77.02, p<0.0001; WO F=3.80, p=0.053. ****p<0.0001. Within each experimental setup, data were normalized to the mean of the control group and expressed as mean ± SEM.

**Supplementary Figure 5. Acute ketamine treatment of primary cortical neurons reduces the expression level of P-T286-CaMKIIa and P-S9-Synapsin**. **a)** Immunoblots and corresponding quantification of P-T286-CaMKIIa/CaMKIIa in primary cortical neurons 30 min upon the treatment with ketamine (100 µM). Data are obtained from 6 independent experiments. Signal for phospho-protein was normalized to total protein and expressed as % of CTRL for each independent experiment. Student’s t-test, ***p<0.001. **b)** Representative immunoblots and quantification of the signal for P-S9-Synapsin/Synapsin 1 from CTRL and ketamine treated cortical neurons (30 min; 100 µM). Data are obtained from 4 independent experiments. Signal for phospho-protein was normalized to total protein and expressed as % of CTRL for each independent experiment and expressed as mean ± SEM. Student’s t-test, *p<0.05. **c)** Correlation analysis of immunofluorescence intensities of P-S9-Synapsin and KCl-evoked syt1-L ab uptake at individual synapses. N=1394 CTRL synapses; N=1479 synapses from ketamine treated cells. The Spearman correlation coefficients for both CTRL and ketamine group are given in the plot. Deming regression fits are shown as black (control) and red (ketamine) lines.

**Supplementary Figure 6. Effect of D-cycloserine (DCS) on KCl-evoked glutamate release *in vivo* and syt1-L ab uptake *in vitro*. a)** Local application of 100 µM DCS into subiculum of wildtype mice significantly reduced KCl-evoked glutamate release. N=7 animals per each group. Student’s t-test, *p<0.05. **b)** Representative images and quantification of KCl-evoked syt1-L ab uptake in CTRL neurons (N=38) and neurons treated 30 min with 10 µM DCS (N=30) and 100 µM DCS (N=34). Statistical significance was assessed using one-way ANOVA followed by Bonferroni’s Multiple Comparison Test; F (2, 99) = 37.73, p<0.0001. **p<0.01, ****p<0.0001. Scale bar 5µm. Within each experimental setup, data were normalized to the mean of the control group and expressed as mean ± SEM.

**Supplementary Figure 7. DPCPX counteracts sustained effects of ketamine on KCl-evoked glutamate release *in vivo* and syt1-L ab uptake *in vitro*. a)** Reduction of KCL-evoked glutamate release in subiculum observed 24h upon systemic administration of ketamine (15 mg/kg) was occluded by pretreatment with DPCPX (2 mg/kg; 30 min prior ketamine). Number of animals per group: N=7 vehicle; N=8 ketamine; N=7 DPCPX; N=7 DPCPX/ketamine. Statistical significance was assessed using two-way ANOVA followed by Fisher's LSD; interaction F (1, 25) = 4.95, p=0.035; ketamine F=3.62, p<0.068; DPCPX F=3.354, p=0.079. **p<0.01. **b)** Pre-treatment of primary neurons with DPCPX (1h; 2.5 µM) prevented the reduction of KCl-evoked syt1-L ab uptake in cells treated for 30 min with 100 µM Ketamine 24h after the drugs were washed out (24h WO). N=38 CTRL cells; N=37 ketamine; N=40 DPCPX; N=41 DPCPX/ketamine. Two-way ANOVA with Bonferroni’s Multiple Comparison Test, interaction F (1, 152) = 2.47 p=0.118; DPCPX F=97 p<0.0001; ketamine F=15.58 p=0.0001; **p<0.01, ****p<0.0001. **c)** Pre-treatment of primary neurons with DPCPX (1h; 2.5 µM) prevented the reduction of KCl-evoked syt1-L ab uptake in cells treated for 30 min with 100 µM (2R,6R)-HNK 24h after the drugs were washed out (24h WO). N=33 CTRL cells; N=30 HNK; N=35 DPCPX; N=35 DPCPX/HNK. Two-way ANOVA with Bonferroni’s Multiple Comparison Test, interaction F (1, 129) = 0.735 p=0.393; DPCPX F=70.35 p<0.0001; HNK F=10.14 p=0.0018; *p<0.05, ****p<0.0001. Within each experimental setup, data were normalized to the mean of the control group and expressed as mean ± SEM.

**Supplementary Figure 8. Ketamine and (2R,6R)-HNK mediated reduction of glutamate release in p11 knockout mice is A1R-dependent.** Local application of 100 µM ketamine or (2R,6R)-HNK into subiculum of wildtype (wt) or p11 knockout (p11ko) mice significantly reduced KCl-evoked glutamate release (wt: N=4 vehicle; N=4 Ketamine; N=5 HNK; p11 ko: N=5 animals per each group). Pretreatment of animals with DPCPX (2.5 μM) occluded the effect of both ketamine and (2R,6R)-HNK (wt: N=6 DPCPX; N=4 DPCPX/ketamine; N=4 DPCPX/HNK; p11 ko: N=6 DPCPX; N=4 DPCPX/ketamine; N=4 DPCPX/HNK ). Two-way ANOVA followed by Fisher's LSD; interaction F (5, 44) = 0.256, p=0.934; drug F=16.3, p<0.0001; genotype F=0.252, p=0.628. *p<0.05, **p<0.01, ***p<0.001. Within each experimental setup, data were normalized to the mean of the control group and expressed as mean ± SEM.

**Supplementary Figure 9. Fluoxetine mediated reduction of presynaptic activity is A1R-independent.** Quantification of KCL-evoked syt1-L ab uptake in primary cortical neurons treated with 100 μM Fluoxetine (FLX) without or with pre-treatment with A1R antagonist DPCPX (1h; 2.5 μM). N=8 CTRL cells; N=10FLX; N=8 DPCPX; N=9 DPCPX/FLX. Two-way ANOVA with Bonferroni’s Multiple Comparison Test, interaction F (1, 31) = 10.82 p<0.003; FLX F=56.95 p<0.0001; DPCPX F=4.47 p<0.043; *p<0.05, ****p<0.0001. Within each experimental setup, data were normalized to the mean of the control group and expressed as mean ± SEM.

**Supplementary Figure 10. Cannabinoid receptors (CB1R) are not involved in ketamine and (2R,6R)-HNK effect on presynaptic glutamate release and activity.** **a)** Reduction of KCl-evoked glutamate release in mice upon local application of vehicle, ketamine (100 µM) or (2R,6R)-HNK (100 µM) without or with co-application of AM251 (2.5 µM) into subiculum. Number of animals per group: N=10 vehicle; N=8 ketamine; N=8 HNK; N=7 AM251; N=9 AM251/ketamine; N=6 AM251/HNK. Statistical significance was assessed using two-way ANOVA followed by Fisher's LSD; interaction F (2, 42) = 1.63, p=0.207; drug F=21.1, p<0.0001; AM251 F=11.39, p=0.002. *p<0.05, **p<0.01, ***p<0.001**. b)** Representative images and quantification of KCL-evoked syt1-L ab uptake in cells treated for 30 min with 100 µM ketamine in the absence or presence of AM251 (2.5 µM). N=35 CTRL; N=40 Ketamine; N=32 AM251; N=31 ketamine/AM251. The statistical analysis was done using two-way ANOVA followed by Bonferroni’s Multiple Comparison Test; interaction F (1, 134) = 31.11, p<0.0001; Ketamine F=23.76, p<0.0001; AM251 F=0.06, p=0.813. ***p<0.001, ****p<0.0001. Scale bar 5µm **. c)** Representative images and quantification of KCL-evoked syt1-L ab uptake in cells treated for 30 min with 100 µM (2R,6R)-HNK in the absence or presence of AM251 (2.5 µM). N=26 CTRL; N=32 HNK; N=30 AM251; N=32 HNK/AM251. The statistical analysis was done using two-way ANOVA followed by Bonferroni’s Multiple Comparison Test; interaction F (1, 116) = 35.59, p<0.0001; HNK F=28.91, p<0.0001; AM251 F=6.98, p=0.009. *p<0.05, ****p<0.0001. Scale bar 5µm. Within each experimental setup, data were normalized to the mean of the control group and expressed as mean ± SEM.
