# Supplementary figures and images for: Ketamine decreases neuronally released glutamate via retrograde stimulation of presynaptic adenosine A1 receptors
Source: Mol Psychiatry. 2021 Aug 11;26(12):7425–35. doi: 10.1038/s41380-021-01246-3 (PMC8872981; doi:10.1038/s41380-021-01246-3)

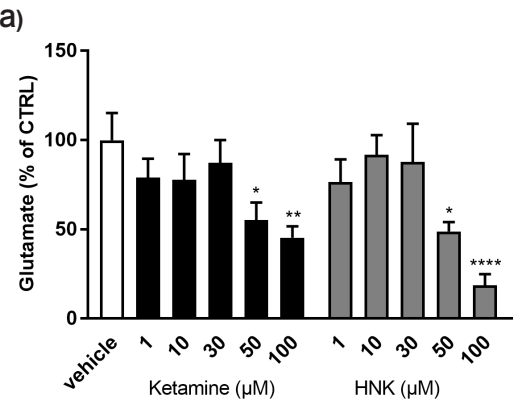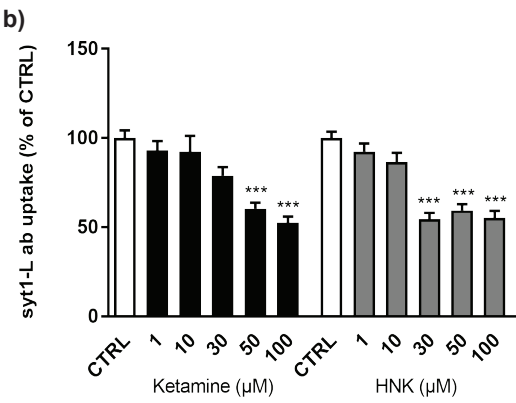

Supplement: Supplementary file 2 — Supplementary Figure 1 [file 41380_2021_1246_MOESM2_ESM.pdf]

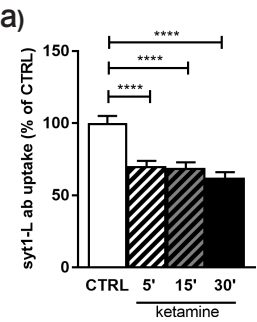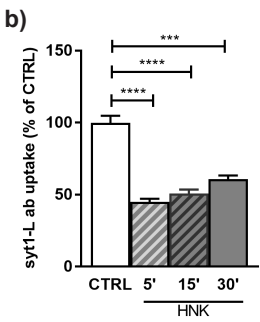

Supplement: Supplementary file 3 — Supplementary Figure 2 [file 41380_2021_1246_MOESM3_ESM.pdf]

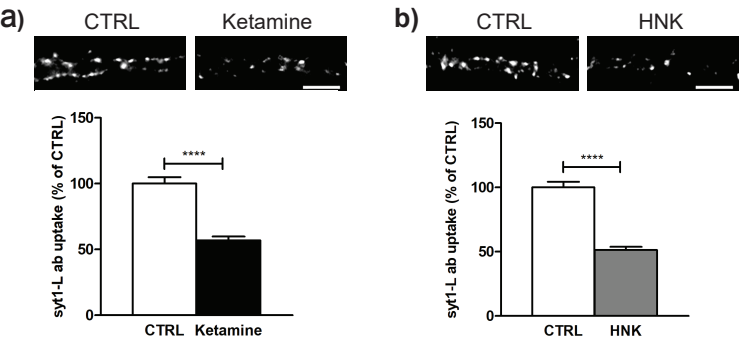

Supplement: Supplementary file 4 — Supplementary Figure 3 [file 41380_2021_1246_MOESM4_ESM.pdf]

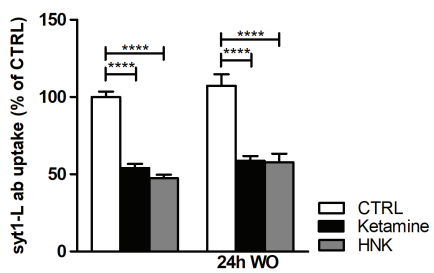

Supplement: Supplementary file 5 — Supplementary Figure 4 [file 41380_2021_1246_MOESM5_ESM.pdf]

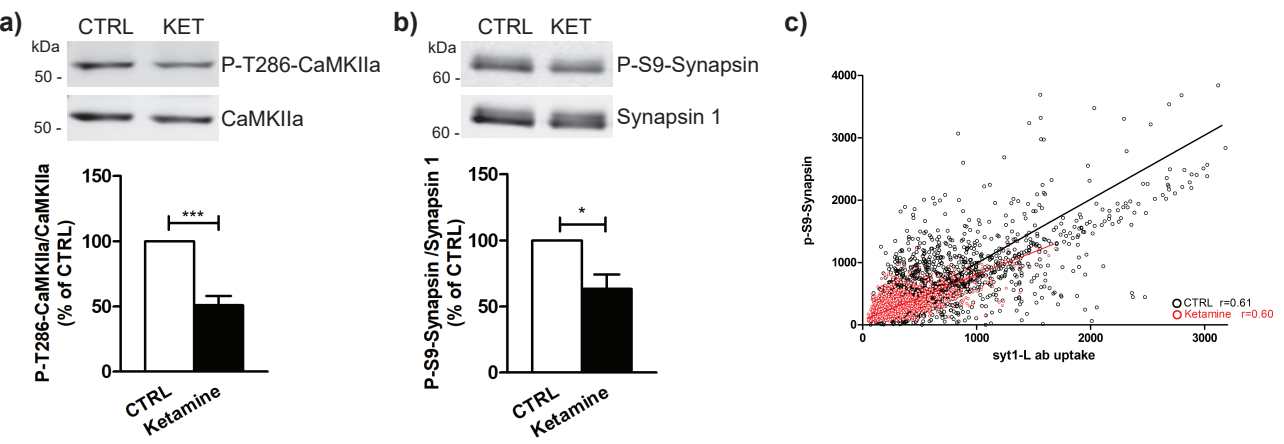

Supplement: Supplementary file 6 — Supplementary Figure 5 [file 41380_2021_1246_MOESM6_ESM.pdf]

a)

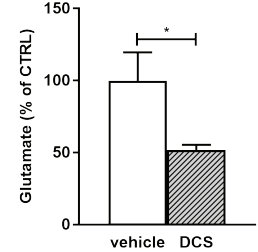

b)

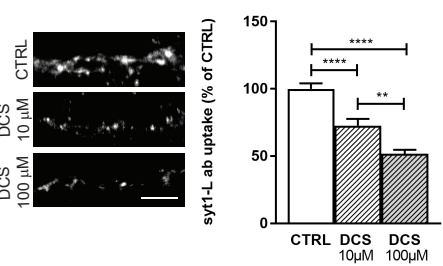

Supplement: Supplementary file 7 — Supplementary Figure 6 [file 41380_2021_1246_MOESM7_ESM.pdf]

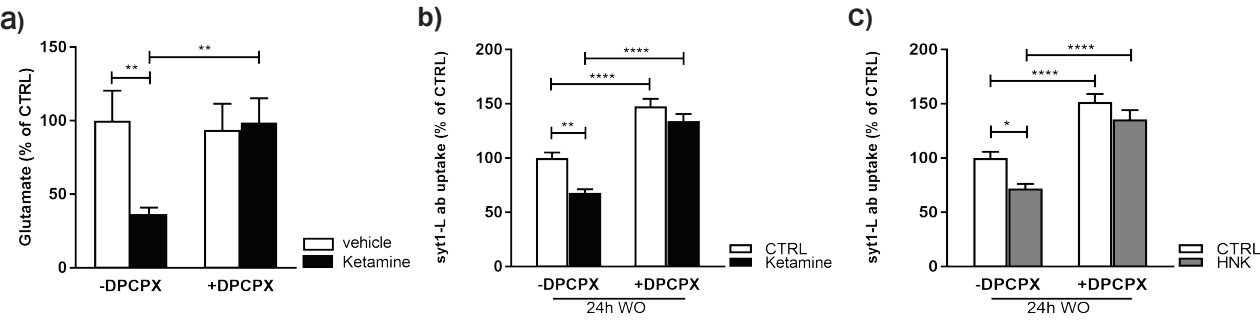

Supplement: Supplementary file 8 — Supplementary Figure 7 [file 41380_2021_1246_MOESM8_ESM.pdf]

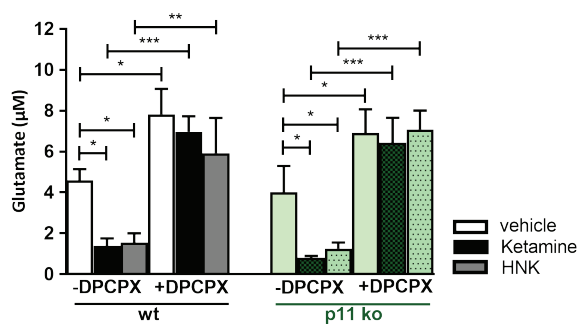

Supplement: Supplementary file 9 — Supplementary Figure 8 [file 41380_2021_1246_MOESM9_ESM.pdf]

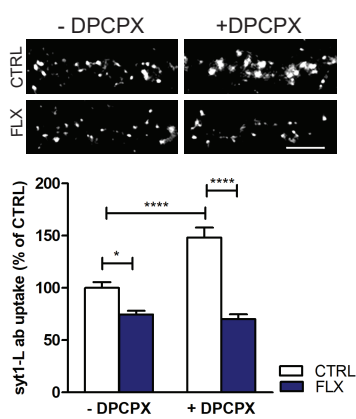

Supplement: Supplementary file 10 — Supplementary Figure 9 [file 41380_2021_1246_MOESM10_ESM.pdf]

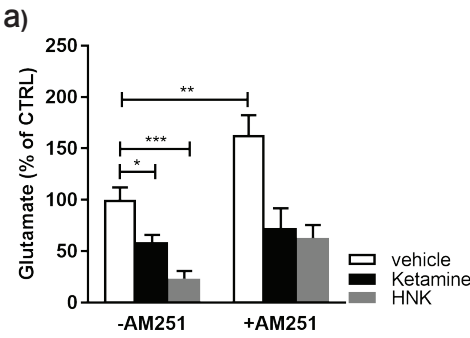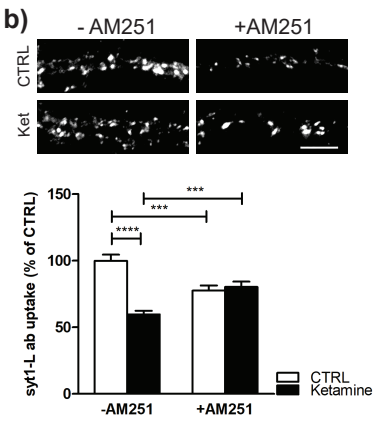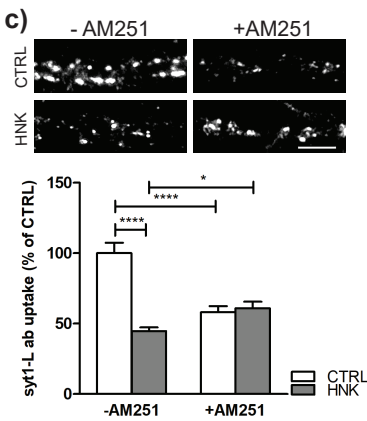

Supplement: Supplementary file 11 — Supplementary Figure 10 [file 41380_2021_1246_MOESM11_ESM.pdf]
